# Effects of a Mental Warmup on the Workout Readiness and Stress of College Student Exercisers

**DOI:** 10.3390/jfmk4030042

**Published:** 2019-06-28

**Authors:** Judy L. Van Raalte, Britton W. Brewer, Allen E. Cornelius, Mary Keeler, Christyan Gudjenov

**Affiliations:** 1 Department of Psychology, Springfield College, Springfield, MA 01109, USA; 2 College of Health Sciences, Wuhan Sports University, 461 Luoyu Road, Wuhan 430079, China; 3 School of Psychology, Fielding Graduate University, 2020 De La Vina Street, Santa Barbara, CA 93105, USA

**Keywords:** exercise, mental preparation, psychological skills, goal setting, imagery, arousal control

## Abstract

The importance of warming up prior to sport competition has been highlighted in the scientific literature, with increasing attention paid to the benefits of mental warmups. The purpose of this research was to explore the possibility that a mental warmup may also benefit exercisers. Two studies were conducted in which the effects of a mental warmup on the psychological readiness and psychological stress of exercisers were examined. Study 1 used a pretest–posttest design and Study 2 used an experimental pretest–posttest design, comparing mental warmup participants to a control group. In both studies, exercisers were assessed before and after they completed a prerecorded mental warmup that consisted of goal setting, imagery, and arousal control. Overall, the results showed that completing a mental warmup increased exercisers’ readiness to exercise and to use mental skills to enhance workouts. The mental warmup also reduced stress. These findings suggest that mental warmup strategies that facilitate readiness for sport performance may have utility in exercise settings. Future research exploring the applicability of a mental warmup in diverse settings, as a stress reduction, and as a potential injury reduction intervention is warranted.

## 1. Introduction

Decades of research have demonstrated the beneficial physical and mental health effects of exercise for people of various ages, race/ethnicities, and socioeconomic levels [1,2]. Guidelines to educate policy makers, medical professionals, and the general public about the amount and type of exercise needed to reduce susceptibility to noncommunicable disease, improve health, and prevent premature death have been created and widely distributed [1,3,4,5]. Although the mental and physical health benefits of aerobic and muscle strengthening exercise are clear, the majority of adults (78%) fail to meet guidelines for such exercise [6].

Not surprisingly, negative feelings about exercise have been found to be related to reduced exercise behavior. For example, lack of motivation is associated with low levels of exercise behavior [7]. Similarly, psychological stress tends to be associated with reduced exercise [8]. That is, people reporting high psychological stress on stress questionnaires, on life event scales, and in diaries tend to exercise less than people not experiencing high stress. People experiencing objectively stressful events such as final exams or the cancer diagnosis of a child tend to exercise less than matched controls (i.e., less stressed populations) and less than they did in less stressful periods [8,9]. Although some stressors (e.g., new romantic relationships, retirement, major achievements, distressing harassment) are related to increased exercise behavior, Stults-Kolehmainen and Sinha [8] concluded that, overall, high levels of psychological stress are associated with lower levels of exercise. Further, they suggested that factors and interventions that reduce stress are likely to have a beneficial effect on exercise.

In the sport psychology literature, researchers have studied the performance of athletes in stressful, competitive environments, with particular attention paid to the psychology of ideal performance states and optimal performance [10]. Ideal performance states have been described as occurring when athletes feel motivated, energized yet relaxed, focused on the task at hand, and have positive attitudes about performance [11]. Perhaps to help reach ideal performance states, elite athletes have been found to regularly use mental preparation skills and to do so more than novice athletes [12]. Among elite athletes, more successful elite (Olympic) athletes have reported greater use of mental preparation skills such as imagery and emotional control prior to competition than have less successful elite athletes [13]. In line with self-determination theory [14] that highlights the value of competence and autonomy, athletes of diverse ability levels have also been found to profit from using psychological skills interventions (for a meta-analysis, see [15]). Based on these findings, specific recommendations related to mentally warming up for sport competition have been developed that include the use of goal setting, imagery, confidence building, arousal regulation (calming down or energizing), and attentional focus [16]. The purpose of mental warmups is to increase athletes’ readiness to use mental skills, including the skills that are part of their usual mental skills training [17]. Similar recommendations have been made for musicians, whose development of mental warmup skills such as self-talk have been found to be related to enhanced self-efficacy and a greater sense of control over some of the debilitating aspects of stress and anxiety [18].

Factors associated with ideal performance states in competitive athletes and musicians such as goal setting appear to overlap with factors related to increased exercise behavior [19,20]. Therefore, it seems possible that the benefits of a mentally warming up demonstrated in the sport psychology literature may extend to exercisers. Specifically, exercisers who identify their exercise-related goals and use imagery, arousal regulation, and attentional focusing skills prior to exercise may become prepared to use these mental skills to workout. Further, exercisers who use these skills during a mental warmup may experience reduced stress, as mental warmup skills (e.g., arousal regulation) have been shown to be stress-reducing [21].

The purpose of this research was to replicate the work of Brewer et al. [17] by examining the effects of a mental warmup on exercisers and extend the work of Brewer et al. [17] by exploring the effects of a mental warmup on exercisers’ psychological stress. Based on previous research with competitive athletes and musicians, it was hypothesized that exercisers who use a mental warmup would become more psychologically ready to exercise after completing the mental warmup [12,13,17,18]. Because psychological skills use has been found to be associated with reduced/manageable stress, it was hypothesized that the mental warmup would also reduce exercisers’ levels of stress [8,18,21]. Reducing stress is important because high stress levels have been associated with unhealthy behaviors [8] and injury [22,23].

## 2. Materials and Methods

This investigation was conducted in accordance with the Declaration of Helsinki. The Springfield College Institutional Review Board gave initial approval to the procedures used in this research on 13 October 2017 under the title “Effects of a Mental Warmup on the Readiness, Stress, and Workout of College Students.” Prior to their involvement in the research, all participants gave their informed consent for inclusion.

### 2.1. Experiment 1

#### 2.1.1. Participants

Participants were 44 college students (*n* males = 17, *n* females = 27) enrolled in psychology courses who self-identified as exercisers. Participants were an average of 19.27 (SD = 1.11) years of age and included first year students (*n* = 16), second year students (*n* = 17), third year students (*n* = 9), and fourth year students (*n* = 2).

#### 2.1.2. Instruments

Two questionnaires were used in this study. The Wellness Center Research Questionnaire consists of demographic items and questions pertinent to exercise experience and plans. The Workout Readiness Questionnaire is adapted from previous research [17] such that the workout readiness items relate to exercise rather than sport and include an item pertaining to stress. The Workout Readiness Questionnaire includes 18 items that assess the state of psychological readiness to perform physical activity and use psychological skills including: (a) the 7-item Psychological Readiness to Perform scale that addresses respondents’ *readiness to perform exercise* (i.e., the extent to which they are prepared, energized, calm, motivated, focused, thinking, and confident) with responses given on a Likert-type scale ranging from 1 (strongly disagree) to 7 *(*strongly agree) and high scores indicating greater readiness; (b) the 6-item Readiness to Use Psychological Skills scale that address respondents’ *readiness to use imagery, focusing skills, motivation skills, thoughts, calming skills, and energizing skills,* scored on a scale from 1 (not at all ready) to 7 (very ready); (c) the 3-item Mental Readiness Form [24] that assesses thoughts, how the body feels, and emotions/feelings on 11-point scales from calm to worried, relaxed to tense, and confident to scared, respectively (all items on this scale were reverse scored and averaged so that a high score reflected high mental readiness to perform); (d) a stress item to rate overall level of stress from 1 (very low) to 11 (very high); and (e) an overall mental warmup item that states, “I am mentally warmed up to perform my best,” rated on a scale from 1 (strongly disagree) to 7 (strongly agree). Adequate internal consistency has been demonstrated for Psychological Readiness to Perform (Cronbach’s alpha values ranging from 0.86–0.91), Psychological Readiness to use Mental Skills (Cronbach’s alpha values ranging from 0.92–0.93) and the Mental Readiness Form (Cronbach’s alpha ranging from 0.74 to 0.87) [17]. The two single-item scales were developed in accord with the suggestion of Tenenbaum, Kamata, and Hayashi [25] for ease of administration in field settings and timely completion of data collection.

#### 2.1.3. Procedure

After receiving institutional review board approval to conduct the study, participants gave informed consent and completed the Wellness Center Research Questionnaire and Workout Readiness Questionnaire. Next, participants stood up, listened to and simultaneously participated in a 5-min prerecorded mental warmup. The 5-min mental warmup audio script was slightly modified from the mental warmup developed by Brewer et al. [17]. That is, the exercise mental warmup omitted information pertaining to having just completed a physical warmup. The mental warmup used for this study is as follows:

Stand tall with your knees bent slightly and your feet shoulders width apart. Breathe through your nose and inhale, filling first the lower part of your lungs, then the middle part, and, finally, the upper part. Hold the breath for a few seconds and exhale slowly, relaxing your abdomen and chest. Take another deep breath through your nose and inhale, again filling first the lower part of your lungs, then the middle part, and, finally, the upper part. As before, hold the breath for a few seconds and exhale slowly, relaxing your abdomen and chest. Resume breathing normally. You can use deep breathing to calm yourself as needed.

Now take a moment to get a clear mental picture of the main thing you want to accomplish in your workout. You can close your eyes as you think about something that is within your control. What do you see in this mental picture of what you want to accomplish? What sensations do you notice in your body? What do you feel in your muscles? What sounds do you hear? What smells and tastes do you notice? Make the mental picture as clear and vivid as you can.

Okay, now let the mental picture fade and focus again on your breathing. Stand tall with your knees bent slightly and your feet shoulders width apart. Breathe through your nose and inhale, filling first the lower part of your lungs, then the middle part, and, finally, the upper part. Hold the breath for a few seconds and exhale slowly, relaxing your abdomen and chest.

Now bring back the mental picture of the main thing you hope to accomplish in your workout today. As the clear and vivid mental picture of what you hope to accomplish reappears, what do you see? What sensations do you notice in your body? What do you feel in your muscles? What sounds do you hear? What smells and tastes do you notice? Allow yourself to fully experience this mental picture, filled with the belief that you can make it happen today.

Let the mental picture fade once again. Imagine a warm glow forming in your stomach, right in your core. This warm glow is full of energy and is slowly starting to spread throughout your body. As the energy spreads, jump up and land with both feet. Shake out your arms and feel the energy starting to surge again from inside you. Feel the energy launch you into the air again, land, and shake out your arms.

Keep that feeling of energy and, as you do, bring back the mental picture of the main thing you hope to accomplish today one final time. Check your energy level. Use the warm glow of energy in your body to raise your energy level or your breathing to find the level of energy you need and get yourself ready to perform. You have the appropriate level of energy, you know what you want to accomplish, you believe you can accomplish it, and you are ready to do it. On the count of three, we will clap our hands three times and then go do it.

Upon completion of the mental warmup, participants again completed the Workout Readiness Questionnaire, were debriefed, were thanked for their participation in the research, and proceeded to their workouts (*M =* 53.13, *SD* = 23.50 min of exercise).

#### 2.1.4. Data Analysis

Pretest responses were used to calculate Cronbach’s alpha for the Mental Readiness Form, the Psychological Readiness to Perform, and Readiness to Use Psychological Skills questionnaires. The distributions of responses all measures were evaluated for suitability for parametric analysis. Paired samples *t*-tests were conducted to determine whether the mean values for all five measures changed significantly from pretest to posttest. All statistical analyses were performed using SPSS version 25.0 (IBM Corp., Armonk, NY, USA).

### 2.2. Experiment 2

#### 2.2.1. Participants

Participants were 84 college students (*n* males = 43, *n* females = 41) enrolled in psychology courses who self-identified as exercisers. Participants were an average of 19.54 (*SD* = 1.24) years of age, across four years in college (*n* first year = 27, *n* second year = 33, *n* third year = 13, *n* fourth year = 11).

#### 2.2.2. Instruments

As in Study 1, the Wellness Center Research Questionnaire was used to assess demographics, exercise experience, and plans and the Workout Readiness Questionnaire was used to assess the state of psychological readiness to perform physical activity and use psychological skills.

#### 2.2.3. Procedure

After receiving institutional review board approval to conduct the study, participants gave informed consent and completed the Wellness Center Research Questionnaire and Workout Readiness Questionnaire.

Participants were randomly assigned to conditions. Participants in the experimental group stood and participated in the same mental warmup used in Study 1 by listening to an audio file. Participants in the control group stood for 5 min to mirror the physical aspect of the experimental group. All participants then completed the Workout Readiness Questionnaire again, were thanked for their participation, were debriefed, and proceeded to their workouts (*M =* 56.57, *SD* = 27.64 min of exercise).

#### 2.2.4. Data Analysis

Pretest responses were used to calculate Cronbach’s alpha for the Mental Readiness Form, the Psychological Readiness to Perform questionnaire, and the Readiness to Use Psychological Skills questionnaire. The distributions of responses all measures were evaluated for suitability for parametric analysis. A 2 (mental warmup, control) × 2 (pretest, posttest) mixed factorial ANOVA was performed for each dependent measure, an approach that facilitates simple effects analyses following statistically significant interactions. All statistical analyses were performed using SPSS version 25.0 (IBM Corp., Armonk, NY, USA).

## 3. Results

### 3.1. Study 1

The Mental Readiness Form, the Psychological Readiness to Perform, and Readiness to Use Psychological Skills questionnaires all had acceptable internal consistency (see Table 1). These values indicate that taking an average of the items is warranted to create a scale. Scale scores were created for both pretest and posttest administrations of each measure (means and standard deviations are presented in Table 1).

As shown in Table 1, paired samples t-tests revealed that mean values for all five measures changed significantly from pretest to posttest indicating that participants who completed a mental warmup were more mentally ready to exercise, to use imagery, focusing skills, motivation skills, thoughts, calming skills, and energizing skills for exercise and were also more calm, relaxed, confident, and less stressed. For the Mental Readiness Form, 86% (*n* = 38) of the sample showed an increase. For Psychological Readiness to Perform, 71% (*n* = 31) of the sample showed an increase. For the Readiness to Use Psychological Skills, 80% (*n* = 35) of the sample showed an increase. For the Stress item, 80% (*n* = 35) of the sample showed a decrease in stress. For the overall mental warmup item, 68% (*n* = 30) of the sample showed an increase.

### 3.2. Study 2

Cronbach’s alpha for the Mental Readiness Form, the Psychological Readiness to Perform questionnaire, and the Readiness to Use Psychological Skills questionnaire all had acceptable internal consistency (see Table 2). Means and standard deviations are presented in Table 2.

The results of 2 (mental warmup, control) × 2 (pretest, posttest) mixed factorial ANOVAs performed for each dependent measure are presented in Table 2. The interaction between time of assessment (pretest and posttest) and experimental condition (mental warm-up or control) was statistically significant for all five dependent variables, indicating the mental warm-up group changed from pretest to posttest in a different manner than the control group. Simple effects analyses were conducted to probe the nature of these interactions. For the Mental Readiness Form, the mental warm-up group showed a significant increase, F(1,43) = 19.14, *p* < 0.001, η_p_^2^ = 0.31, whereas the control group showed no significant change, F(1,39) = 1.53, *p* = 0.22, η_p_^2^ = 0.03. Readiness to Use Psychological Skills demonstrated a similar result, with the mental warm-up group showing a significant increase, F(1,43) = 26.95, *p* < 0.001, η_p_^2^ = 0.38, whereas the control group showed no significant change, F(1,39) = 1.42, *p* = 0.24, η_p_^2^ = 0.03. For the Psychological Readiness to Perform, the mental warm-up group showed a significant increase from pretest to posttest, F(1,43) = 9.73, *p* = 0.003 η_p_^2^ = 0.18, and interestingly, the control group showed a significant decrease, F(1,39) = 4.42, *p* = 0.04, η_p_^2^ = 0.10. For the stress assessment, both groups showed a significant decrease in stress from pretest to posttest, but the decrease for the mental warm-up group was more dramatic, F_control_(1,38) = 7.66, *p* = 0.009, η_p_^2^ = 0.49; F_Mental Warm-up_ (1,42) = 40.16, *p* < 0.001, η_p_^2^ = 0.17.

## 4. Discussion

The purpose of this research was to explore the hypothesis that exercisers who use a mental warmup become more psychologically ready to exercise. The results indicated that after completing the mental warmup, participants rated themselves as more ready to perform, more ready to use psychological skills, more ready in terms of thoughts, body, and feelings, and more mentally warmed up overall. These results are in line with previous theorizing and research with athletes and musicians that highlight the benefits of mental warmups that include goal setting, imagery, confidence building, arousal regulation (calming down or energizing), and attentional focus [12,13,16,17,18]. These findings are novel, however, in that they have been applied to exercisers.

In addition to becoming more mentally prepared to exercise, the participants in Study 1 also reported a reduction in their stress levels after completing the mental warmup. This stress reduction was particularly strong for those participants who began the study with high levels of stress. Because high levels of stress have been related to low levels of physical activity [8], interventions that reduce stress may help people maintain or enhance their exercise behavior. Further, stress has been strongly related to athletic injury [22,23]; so, reducing stress could help reduce injury rates.

Although the results of Study 1 were promising, the pretest–posttest design used in Study 1 did not include a control group. Participants in the study may have been affected by maturation or time effects such that as time passed and participants were getting closer to beginning their exercise activities, their stress decreased and their mental readiness to exercise increased. To provide a more rigorous test of the mental warmup and to explore the replicability of the findings of Study 1, an experimental design with control group was employed in Study 2. The results of study 2 indicated that mental warmup participants reported significantly greater reductions in stress and improvements in mental readiness, readiness to work out, and readiness to use mental skills to enhance the workout from pretest to posttest than did the control group. Overall, the results show that the benefits of mentally warming up extend to the exercise domain and a brief mental warmup increases readiness to work out and reduces stress prior to exercise. Further, the combined results demonstrate the replicability of the effectiveness of a mental warmup in exercise settings with both pre-post and experimental controlled research designs.

The mental and physical health benefits of exercise are well known. However, the majority of adults fail to meet exercise guidelines [6]. The disconnect between expected and actual human exercise behavior, may occur in part because rational processes are not the sole determinant of human behavior [26]. In many instances, gut feelings and impressions such as “I don’t feel like exercising right now” or “I am too stressed to exercise” can be important components affecting readiness to exercise and exercise behavior [27].

Research with competitive athletes and musicians indicates that mental preparation is one tool that can be used to help people feel ready to perform and reap performance benefits in high stakes, high stress environments [12,13,17,18]. In an investigation featuring competitive athletes as participants, Brewer et al. [17] found that use of a mental warmup was associated with increased readiness to perform and to use mental skills to enhance performance in both pre-posttest and controlled experimental designs. They also found that the use of a mental warmup, even when completed on a daily basis over an entire competitive season, was something that athletes were comfortable with and willing to do. That is, athletes reported that they perceived using a mental warmup before all competitions and training sessions to be acceptable when asked to evaluate the mental warmup at the start, middle, and end of a competitive season.

Although it is possible that exercisers will benefit from a mental warmup in a similar manner to competitive athletes because both exercisers and athletes are physically active, it is also possible that exercisers are meaningfully different from athletes. For example, exercisers must motivate themselves to exercise, as they are not accountable to teammates and coaches. The results of this research suggest that exercisers who complete a mental warmup become more psychologically prepared to exercise and to use mental skills to enhance their workouts in a manner that is similar to that of competitive athletes (Brewer et al., in press). Additional research could explore the extent to which the effects of a mental warmup for exercisers is similar to or different from the effects of a mental warmup for competitive athletes. Inclusion of physiological measures such as heart rate, respiration rate, and galvanic skin response might provide additional insight in the effects of mental warmups.

Another beneficial effect of the mental warmup was that it reduced exercisers’ stress level relative to pre-mental warmup levels of stress and to a control group. The stress reducing effects of the mental warmup have important implications for exercisers because high stress levels have been associated with low exercise behavior. The mental warmup can serve as a stress-reducing intervention, reducing a key barrier to exercise behavior and providing the opportunity for exercisers to reap the additional stress-reducing benefits of exercise itself [28]. The combination of the stress reducing effects of a mental warmup, that may support ongoing exercise behavior, paired with the stress reduction that often follows exercise may synergistically work to overcome some of the barriers to engagement in population-wide healthy exercise behaviors [29,30]. It seems possible that the repeated and regular use of a mental warmup that reduces stress could serve as a microdose of stress management that has injury-protective effects in both athlete and exerciser populations, but this this hypothesis remains to be tested.

Many interventions that are effective in research studies turn out to be limited in their long-term impact because people who complete the intervention activities as part of a research protocol discontinue their involvement after the research study has ended [31]. For example, people may exercise as part of a class or with free access to an exercise facility but discontinue their involvement when the class or free access is no longer available. Because the mental warmup for exercise is an inexpensive intervention that can be accessed via cell phone, recorded, shared, and modified to fit the needs of individual exercisers, an important implication of this research is that the mental warmup has potential beyond that of interventions that require access to expensive resources. Testing the effectiveness of the mental warmup as it relates to ongoing exercise use may be a valuable direction for future research as adaptation/customization has been shown to be a valuable component of effective exercise interventions [32]. Personalization of the mental warmup can allow exercisers to focus on components of the mental warmup that best match their needs and interests.

Although the mental warmup was shown to be effective for exercisers in the present studies, there are also limitations to consider. It was not possible to determine which components of the mental warmup were most effective because the mental warmup includes a microdose of several psychological interventions (i.e., goal setting, imagery, arousal regulation, attentional focus). Future research that focuses on the individual psychological skills embedded in the mental warmup can help determine the extent to which the various mental warmup components contribute to the beneficial effects.

Another limitation of this research is the exclusive reliance on self-report measures. Behavioral measures used in addition to questionnaires can provide researchers and exercisers with a fuller understanding of the effects of a mental warmup on meaningful variables. Questionnaires, such as the single-item measure of stress, allow for ease of administration and timely data collection [25]. Due to the brevity of the single-item stress measure, it was not possible to determine what types of stress were affected by the mental warmup or the mechanisms by which stress was reduced following use of the mental warmup. Further research with a more comprehensive stress measurement tool would allow researchers to determine more about the stress reducing effects and processes of the mental warmup. Further, given the promising stress reducing effects of the mental warmup found with exercisers, and the potential of stress reduction to also reduce injuries, future research exploring the effects of the mental warmup on athlete stress seems warranted.

Finally, the participants involved in this research were all healthy college students who volunteered to exercise and complete the research. Exploration of the effects of the mental warmup with people who reflect a range of familiarity with and willingness to engage in exercise would help determine the generalizability of these findings. It is possible that the mental warmup may be of value in a broad range of exercise settings.

## 5. Conclusions

This research featured two studies designed to explore the effects of a brief mental warmup intervention on readiness to exercise and stress. The results showed that exercisers who completed a mental warmup perceived themselves to be more mentally warmed up, less stressed, and more ready to use mental skills to exercise after completing the mental warmup. Further, exercisers who completed the brief mental warmup reported being more mentally warmed up, less stressed and more mentally ready use mental skills to exercise than exercisers who did not complete a mental warmup. Although more research is needed to confirm these findings, the results of this investigation suggest that strategies that facilitate readiness to perform in sport may also have utility in exercise settings.

## Figures and Tables

**Table 1:** Study 1 Descriptive and Inferential Statistics and Measures of Internal Consistency.

Measure	Cronbach’s Alpha	Pretest	Posttest	Difference [95% CI]	η_p_^2^
		*M (SD)*	*M (SD)*	*M (SD)*	
Mental Readiness Form	0.83	7.67 (2.00)	9.34 (1.51)	1.67 (1.50) *** [2.12, 1.21]	0.56
Psychological Readiness to Perform	0.91	5.52 (1.05)	5.91 (1.28)	0.39 (0.86) ** [0.65, 0.13]	0.18
Readiness to Use Psychological Skills	0.84	5.47 (0.83)	6.08 (0.91)	0.61 (0.83) *** [0.86, 0.35]	0.35
Stress	-	5.45 (2.42)	2.89 (1.89)	−2.57 (2.43) *** [−3.31, −1.83]	0.53
Overall Mental Readiness to Perform	-	5.14 (1.30)	6.09 (1.31)	0.95 (1.27) *** [1.34, 0.57]	0.36

* *p* < 0.05, ** *p* < 0.01, *** *p* < 0.001.

**Table 2:** Study 2 Descriptive and Inferential Statistics and Measures of Internal Consistency.

Measure	Cronbach’s Alpha	Group	Pretest	Posttest	Change	Significance of Interaction (F Value)
			*M (SD)*	M (SD)	M (SD) [95% CI]	
Mental Readiness Form	0.83	Warmup	7.83 (1.97)	8.97 (1.62)	1.14 (1.73) [0.61,1.66]	6.95 **
		Control	7.70 (1.99)	7.96 (1.80)	0.25 (1.29) [−0.16,0.67]	
Psych. Readiness to Perform	0.93	Warmup	5.00 (1.32)	5.41 (1.16)	0.40 (0.86) [0.14,0.66]	13.59 ***
		Control	5.24 (1.20)	5.06 (1.26)	−0.18 (0.55) [−0.35, −0.01]	
Readiness to Use Psych. Skills	0.88	Warmup	5.15 (1.01)	5.71 (1.04)	0.56 (0.72) [0.34,0.78]	7.77 **
		Control	5.02 (1.18)	5.15 (1.26)	0.14 (0.69) [−0.09,0.35]	
Stress	-	Warmup	4.77 (2.38)	3.26 (1.68)	−1.51 (1.56) [−1.99, −1.03]	8.98 **
		Control	4.79 (2.37)	4.23 (2.31)	−0.56 (1.27) [−0.98, −0.15]	
Overall Mental Readiness to Perform	-	Warmup	4.80 (1.65)	5.64 (1.35)	0.84 (1.51) [0.38,1.30]	13.66 ***
		Control	5.12 (1.45)	4.90 (1.53)	−0.22 (1.07) [−0.57,0.12]	

* *p* < 0.05, ** *p* < 0.01, *** *p* < 0.001.
